# Powering Agriculture IoT Sensors Using Natural Temperature Differences Between Air and Soil: Measurement and Evaluation

**DOI:** 10.3390/s24237687

**Published:** 2024-11-30

**Authors:** Kamil Bancik, Jaromir Konecny, Jiri Konecny, Miroslav Mikus, Jan Choutka, Radim Hercik, Jiri Koziorek, Dangirutis Navikas, Darius Andriukaitis, Michal Prauzek

**Affiliations:** 1 Department of Cybernetics and Biomedical Engineering, VSB—Technical University of Ostrava, 17. listopadu 2172/15, 708 00 Ostrava-Poruba, Czech Republic; kamil.bancik@vsb.cz (K.B.); jaromir.konecny@vsb.cz (J.K.); jiri.konecny@vsb.cz (J.K.); miroslav.mikus@vsb.cz (M.M.); jan.choutka@vsb.cz (J.C.); radim.hercik@vsb.cz (R.H.); jiri.koziorek@vsb.cz (J.K.); 2 Department of Electronics Engineering, Faculty of Electrical and Electronics Engineering, Kaunas University of Technology, K. Donelaicio g. 73, 44249 Kaunas, Lithuania; dangirutis.navikas@ktu.lt (D.N.); darius.andriukaitis@ktu.lt (D.A.)

**Keywords:** energy harvesting, environmental monitoring, IoT, smart agriculture, temperature measurement, thermoelectric generator

## Abstract

As the need to monitor agriculture parameters intensifies, the development of new sensor nodes for data collection is crucial. These sensor types naturally require power for operation, but conventional battery-based power solutions have certain limitations. This study investigates the potential of harnessing the natural temperature gradient between soil and air to power wireless sensor nodes deployed in environments such as agricultural areas or remote off-grid locations where the use of batteries as a power source is impractical. We evaluated existing devices that exploit similar energy sources and applied the results to develop a state-of-the-art device for extensive testing over a 12-month period. Our main objective was to precisely measure the temperature on a thermoelectric generator (TEG) (a Peltier cell, in particular) and assess the device’s energy yield. The device harvested 7852.2 J of electrical energy during the testing period. The experiment highlights the viability of using environmental temperature differences to power wireless sensor nodes in off-grid and battery-constrained applications. The results indicate significant potential for the device as a sustainable energy solution in agricultural monitoring scenarios.

## 1. Introduction

The rapidly expanding technology and application of the Internet of Things (IoT) serve as a source of innovation for solutions that address challenges in agricultural and environmental monitoring, particularly with the extensive deployment of IoT sensors [1]. These application domains commonly face issues such as complicated deployment [2] and operation in harsh environments [3]. Innovative energy-harvesting technologies offer promising avenues of study to address these issues [2].

Powering an IoT sensor node with a battery has several drawbacks: limited battery life, the need for regular system maintenance, environmental concerns associated with batteries, and the high costs associated with battery implementation [4]. The energy-harvesting paradigm offers a potential solution to these limitations, but it also introduces numerous research challenges [5] because energy-harvesting sources are stochastic and vary according to the type of energy source involved [6]. The challenges include developing optimal energy-harvesting strategies for sufficient energy collection, producing appropriate mechanical designs for specific environmental conditions, and managing the overall cost-effectiveness of systems [7]. Additionally, the variability in harvested energy can be mitigated with intelligent algorithms [8] for smart energy management systems [9], optimized edge computing methods [10], and adaptive measurement techniques [11].

**Figure 1:**
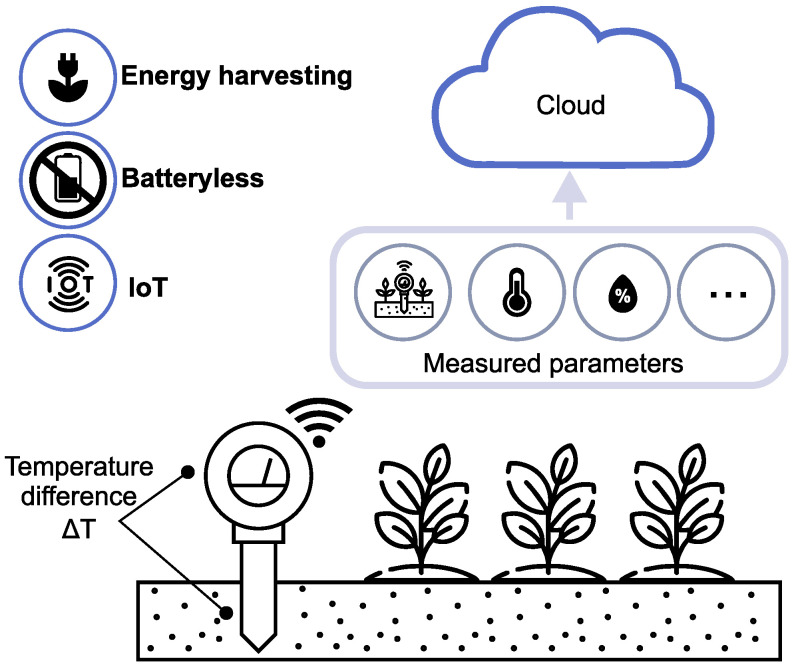
Concept of an energy-harvesting IoT sensor powered by the natural temperature differences between air and soil. Batteryless sensors are powered with harvested energy, and measurement data are transmitted to the cloud.

The current study presents a proposed design for a batteryless IoT sensor node and energy-harvesting subsystem for use in agricultural or environmental applications. The device derives energy from the temperature differences in the environment, using a thermoelectric generator (TEG). A TEG is a solid-state device that converts temperature differences directly into electrical energy based on the Seebeck effect. The Seebeck effect occurs when a temperature gradient is applied across two dissimilar conductive materials, resulting in the generation of a voltage proportional to the temperature difference [12]. Figure 1 illustrates the concept of an IoT sensor node that derives energy from the natural temperature differential existing between air and soil. The electrical energy produced in the TEG powers an autonomous IoT sensor that monitors environmental parameters such as soil moisture, temperature, humidity, and solar irradiance. The IoT node employs intelligent algorithms to adaptively manage available energy and optimize data measurement and transmission to the cloud.

The research presented in this study is significant for its long-term evaluation of an energy-harvesting subsystem designed to exploit low-temperature differentials as a power source for an IoT sensor node. This approach enables a new generation of environmentally friendly, batteryless, maintenance-free IoT sensors. However, the success of the design critically depends on the energy-harvesting subsystem providing a sufficient energy yield. This paper discusses the following contributions in this area:

1. An extensive in-field evaluation of an energy-harvesting design that exploits the low-temperature differentials between air and soil.
2. An analysis of an energy-harvesting design that incorporates multiple temperature sensors to improve future sensor architecture.
3. An evaluation of an energy-harvesting model that uses a DC/DC converter to produce the available energy for IoT sensor operation.

This article is organized as follows: Section 2 reviews and categorizes other proposed solutions (according to their designs), which use temperature differentials as energy sources; Section 3 describes a newly developed prototype energy-harvesting device, the instruments used for monitoring its parameters, data collection during operation, and the calibration and deployment of its equipment. Section 4 presents the results of the experiment; Section 4 presents the results of the experiment; Section 5 discusses temperature in relation to the power generated for the device, the application of measured data to improve the energy harvester’s design, and the suitability of the proposed solution for operation with an IoT sensor node; Section 6 summarizes the experiment and its limitations and suggests future research directions for developing energy-harvesting techniques that can be implemented with IoT devices.

## 2. Related Work

Sensors are increasingly deployed in agricultural environments to collect soil parameter data and improve crop yields. These types of sensors benefit from being maintenance-free. Energy-harvesting methods that use TEGs offer an alternative to photovoltaic cells, which can be obstructed by growing crops.

In [13], the authors investigated energy harvested from soil temperature differences. The experiment measured temperatures at a range of soil depths over a long period and the average temperature differences (ΔT) that occurred between the ground’s surface and each soil level.

Table 1 compares studies that have investigated energy-harvesting systems utilizing temperature differences to generate power. A subsequent study [14] expanded these measurements to an energy-harvesting device of a similar design. Building on previous research, the authors of [13] validated a practical energy-harvesting device and suggested using a thermal gradient at a depth of 30 cm. Conducted over 28 months in Northern Germany, the study reported on the total energy harvested, highlighting the device’s effectiveness. The sensor completed 237,474 tasks, and each task consumed an average of 1.77 mJ. The average positive ΔT at the TEG was 1.70 °C; the negative ΔT was 0.62 °C. The temperature sensor’s precision was within 0.5 °C.

The authors of [15] designed an energy-harvesting device that uses solar radiation as a heat source to collect energy from environmental temperature differences. The device incorporates an aluminum block measuring 500 × 300 × 300 mm, housed in a PVC basin. Nine TEG modules are arranged in a 3 × 3 matrix at the top of the block to cover the entire heat exchange area and they are connected in series. Aluminum heat sinks are positioned at the top of the TEG modules and at the base of the block to form heat exchangers. This setup successfully harvested an average of 6.79 mW during the day and 1.27 mW at night. However, the cost of the device is a major drawback, mainly due to the large mass of the aluminum block, which facilitates heat transfer between the soil’s surface and the surroundings.

In [16], the authors tested an energy-harvesting device of their own design to power a wireless sensor node. The design optimizes heat flow within the device’s structure to minimize losses due to high thermal resistance. The authors discussed various designs and methods to reduce heat loss. Field deployments of the device yielded an average power output of 94 µW to 369 µW. The authors also discussed the device’s power generation efficiency and the need to incorporate an energy management system.

The authors of [17] introduced a gravity-assisted pipe with a heat exchanger at its lowest point. The two experimental devices each contained a heat pipe, 40 mm in diameter and available in lengths of 2.5 m or 3.5 m. Equipped with eight TEGs (TG12-6-02, Marlow Industries Inc., Dallas, TX, USA), the devices generated an average power of 0.335 µW and a peak power of 3.7 µW in the city of Harbin, and an average power of 0.076 µW and peak power of 2.3 µW in Beijing. A notable disadvantage of this design is its length and the material costs.

In [18], the authors described an energy-harvesting device that collects solar energy via a heat sink buried in the soil. The heat sink was deployed at a depth of 10 cm in several locations and harvested a daily average of 24.38 J, which corresponds to an average power of 0.28 mW.

TEGs also find use for energy-harvesting purposes in a range of other applications that exploit heat sources. In [19], the authors described a TEG-powered system for use with critical applications in avionics, motorsports, and aerospace. The system draws from waste heat and the surrounding environment, is capable of transmitting data every 488 ms, and produces a ΔT as low as 10 °C. Another system described in [20] harvests energy from a pipeline to power a wireless sensor node (WSN). A flexible TEG measuring 140 mm × 113 mm derived around 272 mW from a pipe with a temperature of 70 °C. Reference [21] explored the use of asphalt pavement for energy harvesting and succeeded in producing an average of 10 mW of electrical energy. In another study, the authors of [22] discuss the use of volcanic gases and steam for energy harvesting, with the experiment yielding outputs of 0.32 to 0.33 W per deployed module.

## 3. Methods and Experiment

To exploit the natural thermal gradient between soil layers and the ambient air at the ground surface, we constructed a prototype energy-harvesting device. The components are described in Section 3.1. The prototype is equipped with sensors to measure temperature at designated points, data acquisition cards, a chassis, and a single board computer (SBC) for recording and logging measured values in the LabVIEW 2021 application. The measurement instrument parts are described in Section 3.2. Before deployment of the prototype, the temperature sensors were calibrated in a climate chamber to ensure accurate measurement of the temperature differences between soil levels. The calibration process is described in Section 3.3.

### 3.1. Energy Harvester Design

The prototype device introduced above exploits the temperature differences between different soil layers and the environment at the soil surface. The device was also designed for easy installation and modification.

TEGs are solid-state devices that harness the Seebeck effect to convert heat flux directly into electrical energy. These devices are composed of numerous n-type and p-type semiconductor elements arranged between electrically insulating layers with thermoelectric properties. The p-type semiconductors are doped to create an abundance of positively charged carriers (holes), resulting in a positive Seebeck coefficient [23]. Conversely, n-type semiconductors are engineered to provide a surplus of negatively charged carriers, giving them a negative Seebeck coefficient. When the two junctions are connected electrically, positive carriers move toward the n-junction, while negative carriers flow to the p-junction, generating an electric current [24].

**Figure 2:**
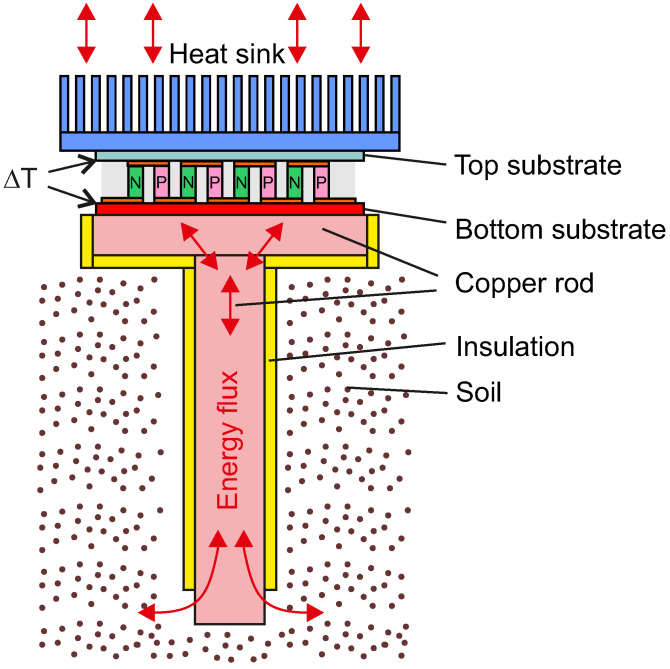
The principle of converting the heat flux generated by the temperature difference between the ground and the ambient air using a TEG.

When one side of the TEG is exposed to a heat source while the other remains cooler, a voltage is generated due to the temperature gradient, as illustrated in Figure 2. The proposed solution uses natural temperature differences between air and soil to establish a directional energy flux, which is then converted into electrical energy through the TEG.

**Figure 3:**
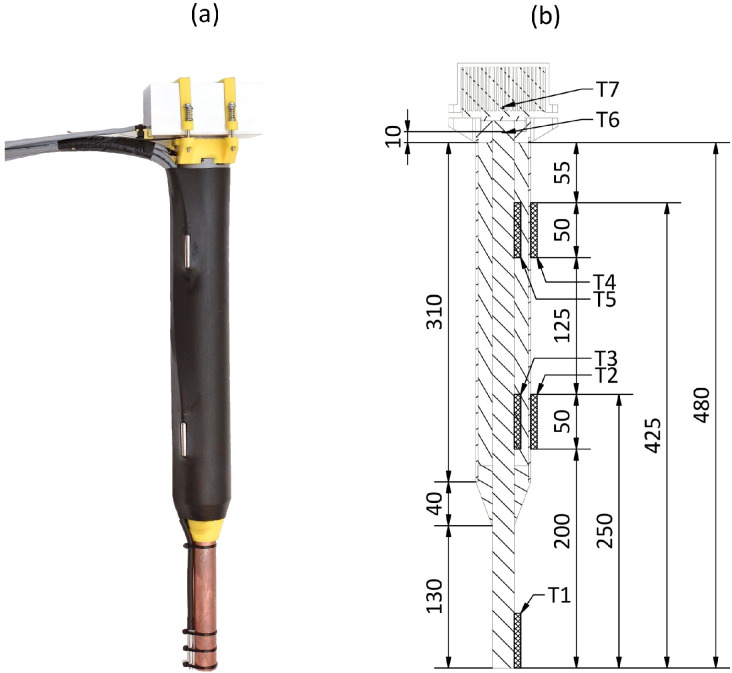
Prototype of energy-harvesting device: (**a**) photograph in profile (**b**) cross-sectional drawing.

Figure 3 depicts the prototype device. The device’s main component is a circular copper rod (ČSN EN 13601 standard [25]) with a diameter of 20 mm and a total length of 500 mm; this size achieves an optimal temperature gradient and minimizes heat loss to the soil. A copper heat distributor is attached at the upper end. The distributor has a square cross-section of 40 mm per side and a thickness of 10 mm, ensuring uniform heat flux across the surface of the Peltier cell to maintain a consistent thermal difference.

The rod was inserted into a 20 mm hole at the heat distributor’s geometric center. To ensure thermal conductivity between the parts, they were held together with the Sn97Ag3 solder (Felder Lottechnik GmbH, Oberhausen, Germany). This assembly was then positioned at the device’s base, which included mounting holes for the heat sink. The base was 3D-printed in high-impact polystyrene for optimal mechanical properties.

The energy-harvesting rod was insulated with polyurethane foam and sheathed in a polypropylene pipe to protect the insulation. A TEG RC12-8-01LS (Marlow Industries Inc. Dallas, TX, USA) was positioned at the top of the heat distributor. The TEG was 40.13 × 40.13 mm in size, with a height of 3.35 mm. For efficient heat transmission, both sides of the TEG were coated with ARCTIC MX-4 thermal paste (ARCTIC GmbH, Zurich, Switzerland). An aluminum heat sink SK 628 100 Al (Fischer Elektronik GmbH, Lüdenscheid, Germany) was mounted to the top of the TEG and secured with clamps of material that was the same as the base.

### 3.2. Measurement Instrumentation

To accurately measure temperature and electrical parameters during the experiment, we used the NI cDAQ platform (National Instruments, Austin, TX, USA).

**Figure 4:**
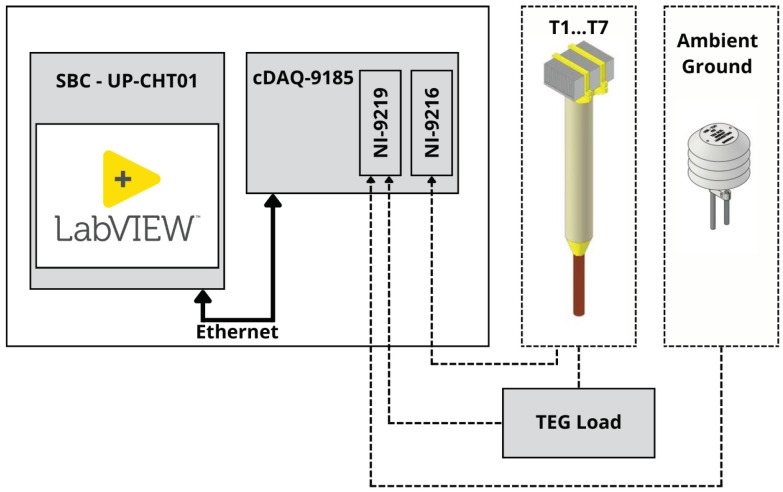
Measurement setup block diagram depicting the data acquisition unit and NI components for collecting temperature data from the prototype.

The system depicted in Figure 4 contains an NI-9216 (National Instruments, Austin, TX, USA) measurement card and PT100 temperature sensors (T1–T7; for specific locations, see Figure 3) for recording temperatures in the energy-harvesting device. The measurement error of the three-wire sensor connection was ±0.20 °C. Two PT100 types were deployed: TT-PT100A-2050-11-AUNI (TEWA Temperature Sensors Sp. z o.o., Lublin, Poland) sensors were positioned above and below the TEG, and 72-23301001-0300.0050.JJ.TM (Gunther GmbH, Schwaig, Germany) sensors were used in all other spots. Both sensor types met tolerance class A with a tolerance of ±0.15 °C. Each sensor was strategically positioned and securely fixed to the device. Thermally conductive glue attached the sensors to metal parts. Sensors not encased in protective capsules were additionally weatherproofed with silicone.

The electrical parameters of the TEG were measured by a NI-9219 (National Instruments, Austin, TX, USA) card. To emulate the TEG’s load, a resistor was used to mirror the TEG’s internal resistance. A small PCB was fabricated for this purpose, containing a 1.5 Ω load resistor and a 0.1 Ω measurement resistor for current measurement. The combined resistance matches the internal resistance of the TEG. The voltage across the measurement resistor and the loaded TEG is recorded by the NI-9219. Additional sensors connected to this card measured the ambient air and ground temperatures. These cards interfaced with the cDAQ-9185 (National Instruments, Austin, TX, USA) chassis, which was subsequently connected to an SBC. Using the LabVIEW programming language, we developed an application to log the device’s measured data.

### 3.3. Measurement Calibration

**Figure 5:**
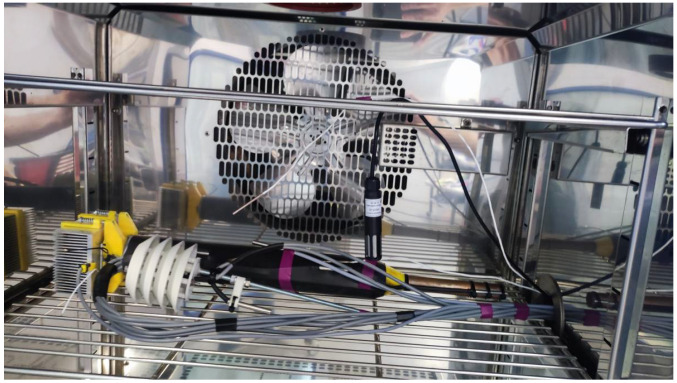
Sensor calibration in a climate chamber to improve temperature measurement accuracy.

For accurate temperature measurement in the prototype’s sensors, we calibrated the device in a CTS C-40/200 climate chamber, which is capable of simulating a temperature range of −40 °C to 180 °C according to user-defined programming. Figure 5 shows the energy-harvesting prototype positioned inside the climate chamber.

The calibration procedure targeted three reference temperatures: −20 °C, 0 °C, and 20 °C. Sufficient time was allocated at each calibration point to allow the energy-harvesting prototype and sensors to stabilize at the target temperature. Temperature readings from all sensors were recorded in LabVIEW from a calibrated temperature data logger equipped with calibrated PT1000 sensors. Individual correction polynomials were then calculated using Equation (1) as follows:

(1) $$p\left(x\right)=p_{1}\cdot x^{2}+p_{2}\cdot x+p_{3}$$

The sensors were calibrated to verify their offsets to the reference measurement points. The measured values were then adjusted according to the polynomials derived for each sensor to obtain optimal accuracy in the temperature differential calculation. The values are presented in Table 2.

### 3.4. Deployment

The experiment was performed in the Czech Republic at coordinates of N $49^{\circ }56.789^{\prime }$, E $18^{\circ }14.379^{\prime }$ m, and an altitude of 215 m. Data were acquired over 12 months, starting in October 2022. During this period, the experiment was interrupted five times for a total of 46 h, producing gaps in the collected data. The energy-harvesting device was installed at a depth of approximately 0.5 m in the soil.

**Figure 6:**
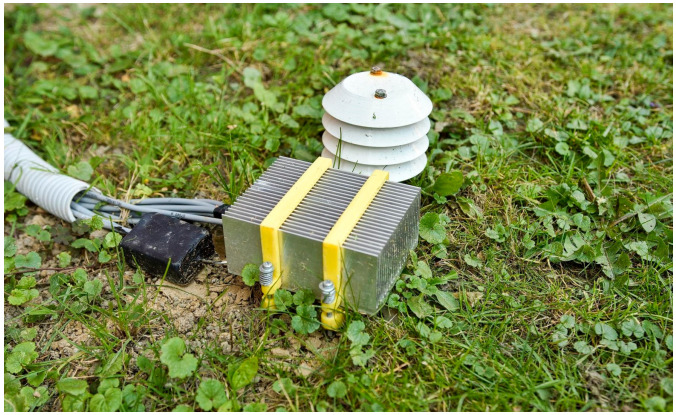
The energy-harvesting device deployed at the experiment site. The upper part of the prototype is shown, with a heat sink and its ambient temperature sensor equipped with a radiation shield.

Figure 6 shows the positioning of the device above ground and the exposure of the heat sink to surface air. Temperature sensors housed inside a solar radiation shield were installed near the energy-harvesting prototype to monitor the agricultural conditions.

## 4. Results

This section discusses the results of the experiment, including details of the amount of energy harvested by the device, the temperature differences measured across the TEG, and corresponding energy generation. This study is committed to ensuring the accessibility and transparency of the research data. In line with this commitment, all the measured data supporting the findings of this research are available on the IEEE DataPort service [26].

**Figure 7:**
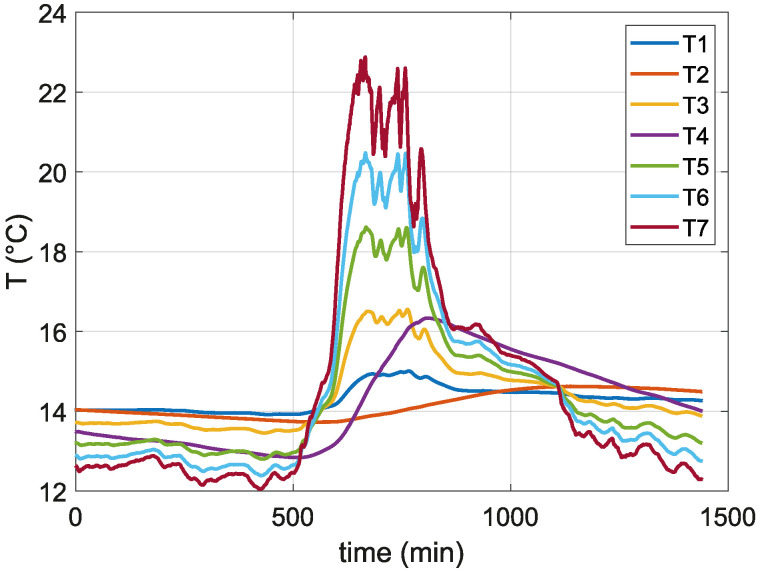
Day temperature curves from T1–T7 PT100 sensors.

Figure 7 presents the temperature measurements recorded over a 24-h period, spanning from midnight of the previous day to the following midnight. It is evident that during the nighttime, temperature T7 is the lowest among all measurements, reflecting its placement on the cooler. Conversely, during the daytime, the influence of ambient temperature causes T7 to become the highest. The other temperatures, T6, T5, T3, and T1, display gradient patterns corresponding to the transition between ambient air and soil. In contrast, temperatures T4 and T2 exhibit a slower rate of change, which aligns with their connection to the soil rather than the copper rod.

**Figure 8:**
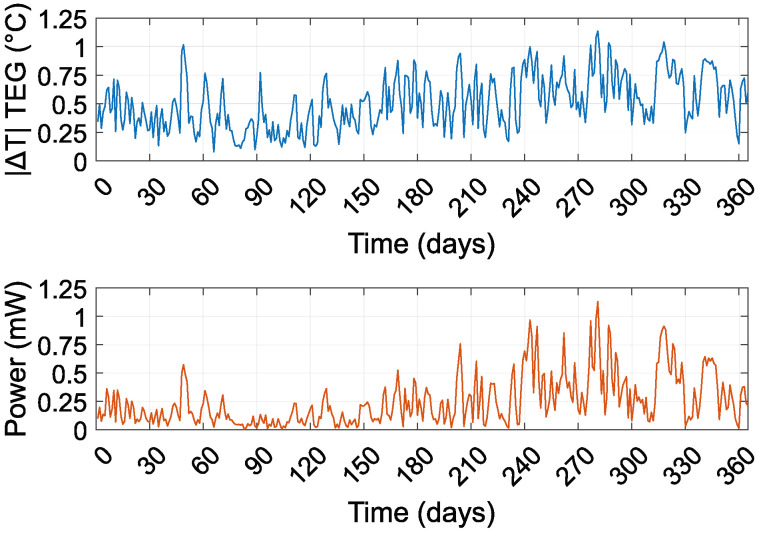
Daily average of the absolute temperature difference and power output on the TEG during the measurement period.

Figure 8 illustrates the daily average absolute temperature difference across the TEG and its corresponding average power output over the course of the experiment. Measurements began in September 2022 (day 0). The results show that the temperature differences during the winter months are generally lower compared to those observed in the summer. The figure also provides an overview of the average temperature difference, which predominantly remains below 1 °C. This limited temperature difference implies that the TEG generates only a very small output voltage under most conditions.

**Figure 9:**
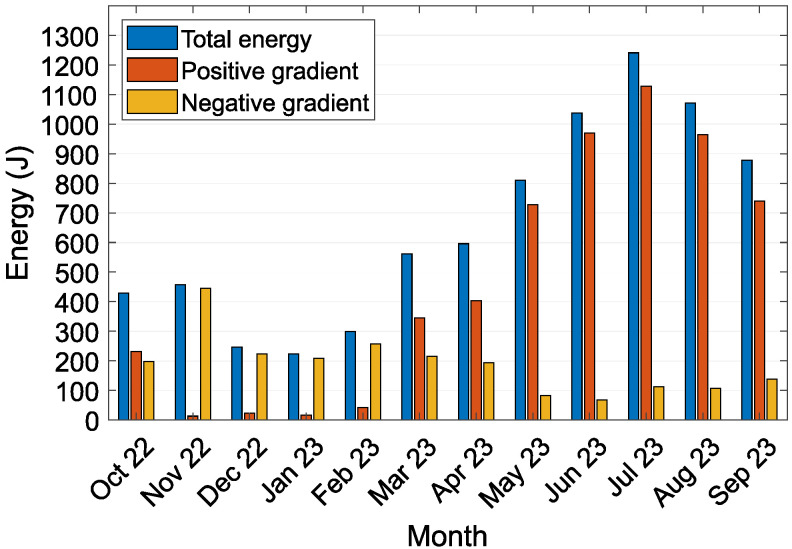
Monthly distribution of energy harvested by the TEG, categorized according to the direction of heat flow through the energy-harvesting device prototype.

Figure 9 plots the total energy harvested by the TEG, divided by the polarity of the temperature gradient. A positive energy gradient indicates thermal energy flowing from the surrounding air to the soil; a negative energy gradient indicates the opposite flow from soil to air. During the measurement period, a total of 7852.2 J of electrical energy was harvested, with 5606.4 J derived from a positive temperature gradient and 2245.8 J from a negative temperature gradient. The monthly energy harvest ranged from 223.6 J to 1241.5 J. As shown in Figure 9, the contributions from negative temperature gradients were significantly greater during the autumn and winter months. During winter, snow cover impeded the energy transfer between the air and heat sink. It is important to note that the energy values were measured directly at the output of the loaded TEG. To estimate the energy available for practical use, we applied a model that simulated the efficiency of an LTC3109 DC/DC converter suitable for extracting electrical energy from the TEG [27].

Naturally, the DC/DC converter is not capable of adjusting the very low voltage from the TEG to a usable voltage without losses. First, there is a threshold of 30 mV, which is the minimum operational voltage of the DC/DC converter. No energy is obtained when the TEG voltage is below this threshold. Moreover, the total efficiency of the DC/DC converter fluctuates between approximately 5% and 30%. For the threshold of 30 mV, the efficiency is 20%, then rises to 30% at 70 mV, and subsequently drops to 5% at 400 mV. The results show that the threshold of 30 mV is a more significant limiting factor for conversion than efficiency. Therefore, to overcome the 30 mV threshold, the temperature difference across the TEG must be at least approximately 1.3 °C [28].

**Figure 10:**
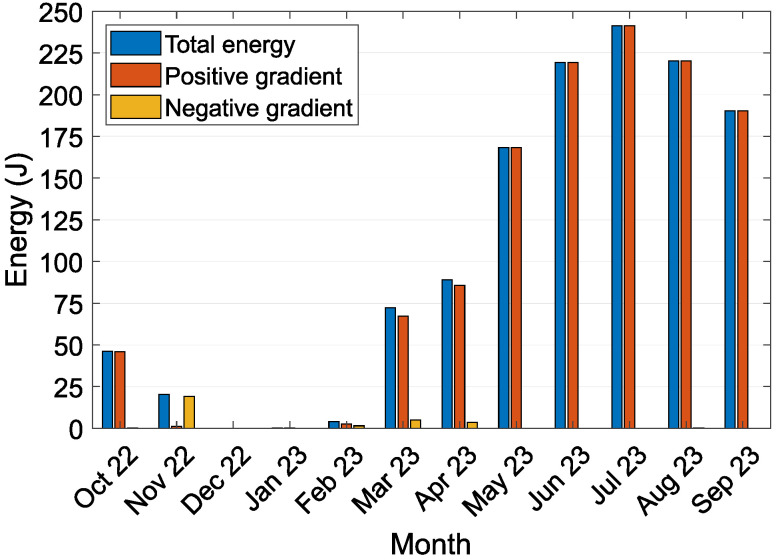
Simulated quantity of energy after conversion by the LTC3109 DC/DC converter according to its efficiency curve, divided by the monthly contributions and heat flow direction through the energy-harvesting device prototype.

Figure 10 shows the simulated quantity of energy after conversion by the LTC3109 DC/DC converter. During the experiment, the energy available per month varied between 0.0 J and 241.4 J, and the total post-conversion quantity of usable energy was 1271.6 J. The highest energy harvest occurred in July 2023. The total energy converted by the DC/DC converter was significantly lower than the potential energy generated by the TEG. However, this energy was sufficient to sustain the operation of the IoT node throughout the year. Unfortunately, during the winter season, the TEG’s output voltage dropped below the required 30 mV threshold, making it insufficient to power the sensor node. Despite this limitation, it did not pose an issue for most agricultural monitoring applications. The average absolute temperature difference on the TEG each month varied from 0.29 °C to 0.71 °C. The smallest temperature difference on the TEG, which indicated the maximum temperature difference during heat transfer from the surface air to the soil, ranged from −0.67 °C to −2.18 °C. Conversely, the largest temperature difference, which indicated the maximum temperature difference during heat transfer from the soil to the surface air, ranged from 1.42 °C to 4.27 °C. The generated average absolute power varied between 0.083 mW and 0.464 mW.

Table 3 provides an overview of the monthly harvested energy and corresponding temperature differences for a one-year period, ranging from October 2022 to October 2023. The table includes key parameters: the total energy harvested (E total) and the energy converted via the DC/DC converter (E DC/DC). Additionally, temperature variations are presented through the maximum (maxΔT) and minimum (minΔT) temperature differences, along with the average temperature difference (|ΔT|). Finally, the table includes the average power (|P|) over the period. The results show a noticeable increase in energy harvested during the warmer months (June to August), peaking in July 2023 with a total of 1241.5 J, which corresponded with the highest average temperature difference (4.12 °C). In contrast, energy harvested in the winter months was lower, particularly in December 2022 and January 2023, where the total energy dropped below 250 J, corresponding with smaller temperature differences.

## 5. Discussion

The discussion is divided into three subsections, each addressing distinct aspects of evaluation and comparison derived from the experimental study. Section 5.1 presents an analysis of the temperature and power parameters. Section 5.2 explores alternative applications of the data with respect to the energy-harvesting prototype’s design. Finally, Section 5.3 examines the challenges and limitations of powering IoT WSNs with harvested energy.

### 5.1. Evaluation of Temperature and Generated Power

The current study aimed to develop an energy-harvesting device for real-world application and assess its capability to exploit temperature differences to power an IoT device.

Table 4 presents a comparative analysis of other experiments that used the natural temperature differences between the ground and air as an energy source. The average observed absolute temperature difference across the TEG ranged from 0.29 °C to 0.71 °C.

Peak temperature differences varied from 1.42 °C to 4.27 °C when heat flowed to the soil and −0.67 °C to −2.18 °C when heat was dissipated through the aluminum heat sink to the air. The average temperature differences are comparable to the studies in [14,15], perhaps because the soil depth for energy harvesting and the design of the harvesting units are similar.

Few studies have reported and compared results for maximum power output, a parameter that depends on the selected TEG module. In the current study, the average absolute power output ranged from 0.083 mW to 0.464 mW, slightly higher than the power output observed in [17], which, significantly, used eight TEG modules. The results reported in [16] indicate notably lower values; however, these are measured post-DC/DC conversion and take into account the efficiency curve of the LTC3109. The authors of [15] reported the highest power output, but when adjusting for the number of TEG modules used in the experiment, the results aligned closely with the findings of the current study. The overall energy harvested in the studies [17,18] and the current study are of a similar magnitude.

### 5.2. Energy Harvester Design

We compared the positioning and number of temperature sensors on the prototype device with simulated models to optimize parameters such as materials and component dimensions. Previous studies have often used expensive materials such as rods and tubes with high thermal conductivity. Optimizing the lengths and shapes of these components allows the desired electrical performance to be achieved and helps to reduce costs. Alternative heat transfer materials could also be explored for even more cost-effective solutions.

The power output of the harvesters can also be scaled for more demanding applications or combined with other ambient power sources to meet the electrical requirements of the device. Optimizations can improve WSN node operation reliability, provide more robust solutions for embedding electronics in the harvester’s design, and enable maintenance-free operation.

One critical consideration for deploying energy-harvesting IoT devices in real-world agricultural environments involves their exposure to harsh environmental conditions. These include being covered by soil, debris, or organic matter during heavy winds and rain. In scenarios where the heat sink or sensors are covered by soil or organic material, the efficiency of heat transfer could be significantly reduced, diminishing the temperature gradient across the TEG and, consequently, the harvested energy. This challenge necessitates the inclusion of debris-resistant designs, such as elevated or shielded heat sinks, self-cleaning mechanisms, or surface coatings that prevent accumulation. These strategies, however, must be balanced against cost and complexity, especially for large-scale deployments in cost-sensitive agricultural operations. Future iterations of the device could incorporate adaptive designs to mitigate these risks. For instance, combining the TEG with alternative energy sources, such as photovoltaic cells, could provide a hybrid solution that ensures consistent power generation despite environmental variability. This approach would address operational challenges, particularly in regions prone to extreme weather conditions.

### 5.3. IoT Node Sensor Operation

IoT or WSN nodes typically contain a sensor for data acquisition, a microcontroller unit (MCU) for data processing, and a wireless module for data transmission. For efficient operation with energy-harvesting sources, these nodes must employ low-power techniques during inactive periods to conserve energy. Cellular technologies, such as general packet radio service (GPRS), are not considered low-power and are therefore not suitable for this specific application.

Table 5 outlines the power consumption of selected WSN nodes used in several studies. Power consumption indicates the energy required for the microcontroller unit (MCU) operation and wireless data transmission. In this context, payload refers to the specific wireless standard employed for data transmission. It should be noted that the volumes of data transmitted, signal strength, and other relevant parameters differ between the selected studies; the table mainly illustrates the approximate quantity of energy required to operate the WSN node. In these studies, energy consumption per WSN cycle ranged from 0.9234 mJ to 124 mJ per transaction.

An examination of the energy consumption data in Table 5 and the total usable energy post-DC/DC conversion (1271.6 J) indicates that the potential data transmission capacity ranged from approximately 10,254 to 1,377,084 messages over a 12-month period. In particular, during the winter, the available energy for consumption was minimal or zero. This highlights the need for energy management strategies, optimized edge computing, and adaptive measurement techniques in conjunction with the proposed device. The application of such approaches in a WSN enables efficient energy use, effective data compression, adjustable measurement intervals, and sufficient reserve energy storage for periods of energy scarcity.

### 5.4. Experiment Findings

In the discussed application, the TEG is capable of generating a certain amount of energy over the course of a year. However, it was observed that the average temperature difference is less than 1 °C, which is insufficient for the DC/DC converter to operate at all. To address this limitation, it would be beneficial to consider connecting multiple TEGs in series to achieve a higher output voltage. Nevertheless, this approach inevitably increases the cost of the IoT node and leads to larger device dimensions, which may impact its feasibility and deployment in space-constrained or cost-sensitive environments.

Given that the DC/DC converter can operate at a temperature difference of approximately 1.3 °C on the TEG, connecting two or three TEGs in series could potentially triple the total voltage output, significantly enhancing the converter’s performance. However, even with a single TEG, the IoT node is capable of limited functionality. Weather fluctuations cause temporary increases in the temperature difference across the TEG, which are sufficient to activate the DC/DC converter and thus enable the IoT node to operate intermittently. This behavior suggests a trade-off between optimizing continuous performance and maintaining a compact, cost-effective design.

## 6. Conclusions

The paper discusses energy-harvesting devices developed to exploit the temperature differential between soil and its surface environment. The paper also reviews state-of-the-art devices that have also used environmental thermal gradients as heat sources. The presented prototype device harnesses these temperature differentials and converts energy through a TEG. The device’s 3D-printed components allow easy assembly and installation. The device includes a comprehensive system for measuring temperatures and the amount of harvested energy. Equipped with several sensors, the prototype was deployed for data collection over a 12-month period.

The experiment yielded a total of 7852.2 J of electrical energy over 12 months. The monthly energy harvested varied from 223.6 J to 1241.5 J, and when factoring in the simulated efficiency of a DC/DC converter, 0.0 J to 241.4 J. The average absolute power generated ranged from 0.083 mW to 0.464 mW. The calculated average monthly absolute temperature difference across the TEG varied from ΔT 0.29 °C to 0.71 °C. When heat transferred to the soil, peak temperatures ranged from 1.42 °C to 4.27 °C; when heat dissipated through the aluminum heat sink to the air, peak temperatures ranged from −0.67 °C to −2.18 °C. The data collected from the prototype offered valuable insight into the temperature gradients experienced by the device and the amount of energy harvested by the TEG. These data are crucial for simulations of parameters and optimizations designed to maximize energy-harvesting efficiency.

The experimental results demonstrate that for most of the year, a single TEG is capable of supplying sufficient energy to power an IoT node, highlighting its potential as a sustainable energy source for agricultural applications. During the winter months, however, the temperature difference is too small for the IoT node to function effectively. This limitation, however, is not a significant obstacle for most agricultural applications, as fields are typically not actively used during the winter season. Furthermore, this study suggests the possibility of connecting multiple TEGs in series, which could address this limitation and enable the technology to function effectively even during colder months.

A limitation of the current study is the absence of a real WSN link to the energy harvester prototype. Future directions for work include the integration of a real WSN in combination with a harvester optimized based on the data collected in this experiment. Future research will test an enhanced harvester design in actual WSN applications and incorporate a sensor node with an energy-efficient wireless module and effective power management strategies. To broaden the applicability of future results, it is essential to deploy the device in multiple locations and factor in the environmental variations affecting the energy harvester.

## Figures and Tables

**Table 1:** Comparison of energy-harvesting systems that exploit the ΔT between soil, air, and other sources of heat.

Study/Experiment	Depth (m)	Main Source of ΔT	TEGs Used	Heat Transfer Medium
Pullwitt [14]	0.3	Air + Soil	1	Aluminum bar
Massaguer [15]	0.5	Solar + Soil	9	Aluminum block
Ikeda [16]	0.3	Air + Soil	1	Copper rod
Huang [17]	2.3 and 3.3	Air + Soil	8	Gravity-assisted heat pipe
Carvalhaes [18]	0.1 and 0.4	Solar + Soil	1	Aluminum bar

**Table 2:** Calibration polynomials derived from the calibration procedure for each sensor channel.

Card + Channel	$p_{1}$	$p_{2}$	$p_{3}$
NI9216—CH0	−0.0008	1.0180	0.3539
NI9216—CH1	−0.0006	1.0157	0.2013
NI9216—CH2	−0.0004	1.0106	0.1657
NI9216—CH3	−0.0001	1.0042	−0.0604
NI9216—CH4	−0.0005	1.0179	0.0934
NI9216—CH5	−0.0003	1.0165	0.0104
NI9216—CH7	−0.0002	1.0176	−0.1324
NI9219—CH0	−0.0006	1.0030	0.6367
NI9219—CH1	−0.0002	0.9992	0.5236

**Table 3:** Monthly harvested energy and temperature differences.

Month	E Total (J)	E DC/DC (J)	Max ΔT (°C)	Min ΔT (°C)	|$\bar{\Delta T}$| (°C)	|$\bar{P}$| (mW)
October 2022	429.1	46.1	2.92	−1.06	0.43	0.160
November 2022	457.6	20.2	1.76	−1.44	0.43	0.178
December 2022	245.7	0.0	1.42	−1.12	0.32	0.096
January 2023	223.6	0.3	1.80	−1.05	0.29	0.083
Febuary 2023	299.1	4.0	2.12	−1.30	0.42	0.124
March 2023	561.1	72.3	3.41	−2.18	0.54	0.210
April 2023	596.0	89.1	4.10	−1.41	0.52	0.230
May 2023	810.0	168.3	3.91	−0.67	0.55	0.311
June 2023	1037.9	219.2	4.27	−0.85	0.63	0.400
July 2023	1241.5	241.4	4.12	−1.03	0.71	0.464
August 2023	1072.0	220.3	4.19	−1.79	0.63	0.400
September 2023	878.6	190.4	3.84	−0.94	0.63	0.339

**Table 4:** Comparison of studies that attempted to harvest energy by exploiting the differences in temperature between the ground and surface air.

Study/Experiment	Average Power Output	Harvested Energy	Achieved Temperature Difference
Pullwitt [14]	-	237,474 tasks (1.77 mJ avg.)	average pos. 1.70 °C/neg. 0.62 °C
Massaguer [15]	7.9 mW	587 J day/110 J night	peak pos. 3.6 °C/neg. 1.2 °C
Ikeda [16]	94–369 µW	-	0.65–1.61 °C (simulation)
Carvalhaes [18]	-	avg. 24.38 J/day	-
Huang [17]	average 0.335 mW/0.076 mW	5209.92 J/1181.95 J (6 month period)	peak 4.62 °C/1.79 °C
Proposed solution	average 0.083–0.464 mW	DC/DC 0.0–241.4 J RAW 223.6–1241.4 J per month	average abs. by month ΔT 0.29–0.71 °C

**Table 5:** Energy consumption comparison in WSN nodes.

Study/Experiment	Energy Consumed	Payload
Pullwitt [14]	1.77 mJ per task avg.	IEEE 802.15.4 frame
Rajab [29]	0.9234–1.3434 mJ	LPWAN sensor node
Lauer [30]	5.90–17.98 mJ	BLE WSN
Bouguera [31]	1.2–115 mJ	LoRa transmission
Orfei [32]	124 mJ	LoRa sensor

## Data Availability

The datasets generated and/or analyzed during the current study are available in the “Temperature and Energy Output Data from a TEG Energy-Harvesting Prototype” repository, https://doi.org/10.21227/1zc0-ry31 (accessed on 26 November 2024).
